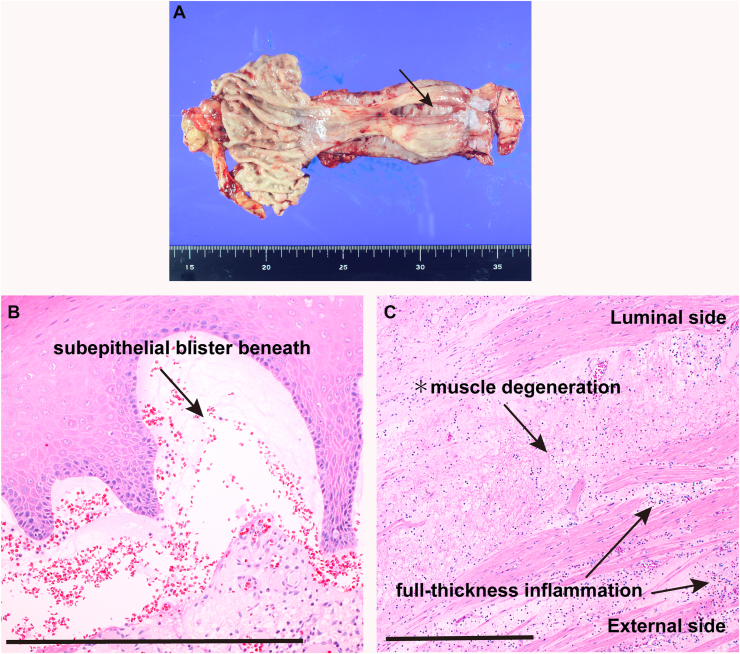# Esophageal Rupture in Recessive Dystrophic Epidermolysis Bullosa

**DOI:** 10.1016/j.gastha.2026.100885

**Published:** 2026-01-17

**Authors:** Yutaro Hara, Takahiro Muroya, Kenichi Hakamada

**Affiliations:** Department of Gastroenterological Surgery, Hirosaki University Graduate School of Medicine, Hirosaki, Japan

A 44-year-old woman with severe generalized recessive dystrophic epidermolysis bullosa (RDEB) developed sudden anterior chest pain while eating. She reported no vomiting or other precipitating events that could increase intraesophageal pressure. Contrast esophagography demonstrated extravasation from the midthoracic esophagus into the right pleural cavity, and computed tomography revealed pneumomediastinum and a right pleural effusion. Emergency thoracoscopic subtotal esophagectomy with retrosternal gastric-conduit reconstruction was performed. Gross examination revealed a 5-cm longitudinal full-thickness perforation in the midthoracic esophagus (Figure A, arrow). Histological evaluation showed a subepithelial blister beneath the stratified squamous epithelium (Figure B, arrow), consistent with RDEB. Full-thickness chronic inflammation (Figure C, arrows) and degenerative changes with disruption of the inner circular muscle layer were also noted (Figure C, asterisk), indicating profound structural fragility of the esophageal wall. These findings support spontaneous esophageal rupture due to intrinsic mural fragility rather than an acute increase in luminal pressure. The postoperative course was uneventful, and the patient was discharged on postoperative day 22. Spontaneous esophageal rupture in RDEB is exceedingly rare and highlights the potential for mural fragility leading to catastrophic full-thickness failure of the esophageal wall in this condition.

**Figure:**